# Supplementary material for: Fast detection of dam zone boundary based on Otsu thresholding optimized by enhanced harris hawks optimization
Source: PLoS One. 2023 Feb 6;18(2):e0271692. doi: 10.1371/journal.pone.0271692 (PMC9901759; doi:10.1371/journal.pone.0271692)
Supplement: S1 Appendix — (DOCX) [file pone.0271692.s001.docx]

**Appendix. Benchmark Functions of Table 3**

1.

2.

3.

4.

5.

6.

7.

**Appendix. Benchmark Functions of Table 4**

1.

2.

3.

4.

5.

6.

7.

8.

9.

10.

11.

12.

13.

14.

15.

16.
