# Supplementary material for: Fast detection of dam zone boundary based on Otsu thresholding optimized by enhanced harris hawks optimization
Source: PLoS One. 2023 Feb 6;18(2):e0271692. doi: 10.1371/journal.pone.0271692 (PMC9901759; doi:10.1371/journal.pone.0271692)
Supplement: Attachment — Submitted filename: Response to Reviewers.doc [file pone.0271692.s002.doc]

Detailed responses to the comments
Manuscript ID: PONE-D-22-03955
Title: Fast Detection of Dam Zone Boundary Based on Otsu Thresholding Optimized by Enhanced Harris Hawks Optimization
Authors: Xiaofeng Qu, Jiajun Wang, Xiaoling Wang, Yike Hu, Tianwen Tan, Dong Kang 

Dear editor and reviewers,
Thank you for your letter and for the reviewers’ comments concerning our manuscript entitled” Fast Detection of Dam Zone Boundary Based on Otsu Thresholding Optimized by Enhanced Harris Hawks Optimization” (PONE-D-22-03955). Those comments are all valuable and very helpful for revising and improving our paper, as well as the important guiding significance to our researches. We tried our best to improve the manuscript and made some changes in the manuscript. The detailed responses to comments are shown as follows: The contents marked in blue represent reviewers’ suggestions and comments, contents marked in red are the revised portion in the revised paper, contents marked in green with strikethrough represent the portion in the manuscript that has been deleted in the revised paper, the yellow highlights represent the explanation of the revision of the manuscript and the black font represents the original portion which is kept in the revised paper. All responses firstly answer the reviewers' comments, then give the specific modification and indicate specific location.
We appreciate for Editors and Reviewers’ warm work earnestly, and hope that the correction will meet with approval.

Responses to the reviewers' comments

Reviewer #1: 
1- Why HHO and no other approaches. This algorithm is a new one so if you want to improve it, you have to compare it to the standard version of the HHSO and utilized the same problems that have been investigated before. Then you can use the standard and improved HHO for your specific field.
Answer：Thank you very much for your comments. HHO has been proved to be a good method in other literatures (literatures 14-18), so we initially selected HHO as the basic method to optimize Otsu (manuscript Page2). I'm sorry for the lack of comparison, which has puzzled you. According to your constructive suggestions, we compared HHO with other algorithms. In the comparison, we found that HHO has better stability and is relatively less likely to fall into local optimal solution. However, HHO still has the possibility of falling into the local optimal solution, so we improve HHO to make it more difficult to fall into the local optimal solution, so as to better meet the complex and changeable site. Therefore, in 3.2 the proposed HHO enhanced by tanh and PSO, we added comparison tables and instructions (manuscript page 4-5). 
Table 1 shows that HHO can find the optimal solution relatively better than AHA[22] and GNDO[23].However, it falls into the local optimal solution in a few cases. This study optimizes the exploration and exploration-development transformation stages and the population initialization. 
Table 1. Comparison of HHO with AHA and GNDO.
Function	Name	d	fmin	HHO	AHA	GNDO
	Sphere Function	30	0	8.67E-84	1.54E-146	8.32E+03
	Schwefel’s Prolem2.22	30	0	2.07E-43	5.79E-66	3.04E+01
	Schwefel’s Prolem1.2	30	0	1.27E-51	7.59E-112	1.19E+04
	Schwefel’s Prolem2.21	30	0	3.45E-40	1.43E-56	3.83E+01
	Generalized Rosenbrock’s Function	30	0	8.66E-02	2.79E+01	3.75E+06
	Step  Function	30	0	5.63E-03	1.15E+00	8.68E+03
	Quartic Function i.e.niose	30	0	3.89E-04	5.92E-04	3.17E+00
	Generalized Schwefel’s Prolem2.26	30	−418.9829×n	-1.26E+04	-1.01E+04	-5.67E+03
	Generalized Rastrigin’s Function	30	0	0.00E+00	0.00E+00	1.25E+02
	Ackley Function	30	0	8.88E-16	8.88E-16	1.51E+01
	Generalized Griewank’s Function	30	0	0.00E+00	0.00E+00	6.56E+01
	Generalized Penalized Function(1)	30	0	6.64E-05	2.05E-02	1.81E+06
	Generalized Penalized Function(2)	30	0	3.93E-04	2.29E+00	1.16E+07
	Shekel's Foxholes Function	2	1	1.64E+00	1.10E+00	2.38E+00
	Kowalik's Function	4	0.00030	4.99E-04	4.15E-04	5.79E-03
	Six-Hump Camel-Back Function	2	-1.0316	-1.03E+00	-1.03E+00	-1.03E+00
	Branin Function	2	0.398	3.98E-01	3.98E-01	3.98E-01
	Goldstein-Price Function	2	3	3.00E+00	3.00E+00	3.00E+00
	Hartman's Family(d=3)	3	-3.86	-3.85E+00	-3.86E+00	-3.86E+00
	Hartman's Family(d=6)	6	-3.32	-2.94E+00	-3.30E+00	-3.16E+00
	Shekel's Family(m=5)	4	-10.1532	-5.50E+00	-7.45E+00	-6.12E+00
	Shekel's Family(m=7)	4	-10.4028	-5.29E+00	-8.14E+00	-6.95E+00
	Shekel's Family(m=10)	4	-10.5363	-5.52E+00	-7.74E+00	-6.95E+00
Error				5	7	18

In addition, we also compare the results in Fig. 4 and Fig. 5.

Fig.4 Convergence behavior for unimodal problems.

Fig.5 Convergence behavior for multimodal problems (F21-F23).
2- Too many references are cited. What are the differences of your work with others?
Answer：Thank you very much for your comments. According to the constructive opinions put forward by you and other experts, we combine the integration and literature review, and replace some of them with the literature recommended by other experts. This not only reduces the amount of references, but also shows the superiority of the method. (manuscript page 1-2).
Conventional threshold segmentation methods, such as the Otsu algorithm [4], can calculate a paving boundary. However, the calculation time is too long to meet the actual needs of the project [5, 6]. The acceleration of threshold segmentation is an optimization problem [7, 8]. Optimization problems have been widely studied in practical engineering applications [9]. The swarm intelligence algorithm has significant computing power and efficiency, and it can overcome the disadvantage of the Otsu algorithm [17]. Swarm intelligence simulates the various group behaviors of social insects or animals and uses the information interaction and cooperation among individuals in a group to achieve optimization. In recent years, numerous swarm optimization algorithms have been proposed and widely applied, such as particle swarm optimization(PSO)[18, 19], grey wolf optimization[20],and Harris hawks optimization(HHO)[21]. Heidari et al. [21] compared HHO with other algorithms and demonstrated its advantages. The superiority of HHO has been demonstrated in numerous applications[22-24]. Therefore, this study uses HHO to optimize the Otsu algorithm to improve the speed while ensuring accuracy. However, the accuracy of HHO is significantly affected by population initialization, and the convergence speed and accuracy cannot meet engineering requirements. In addition, HHO has the disadvantage that it falls into local optimization. Therefore, to solve the problem of population initialization, this study uses the chaotic sine map to optimize the initial population. Owing to the low convergence speed and insufficient convergence accuracy, the tanh activation function is used to optimize the escape energy of prey (threshold). The PSO is used to optimize the exploration stage to address the problem of local optimization. 


 3- Is your work novel? Are there any other related work in the literature that worth mentioning in sec. 2? Please write about the novelty of your work. What are the main challenges in your field?
Answer：Thank you for your constructive comments. According to your comments, I have added a new chapter engineering optimization problems to illustrate the main challenges in the field and the innovation and practicability of this paper. (manuscript page 2-3).
2. Engineering optimization problems
This study focuses on the detection of paving boundaries in the core wall area of earth-rock dams. Images are mainly obtained using the monitoring cameras on the dam abutments on both banks. The conventional Otsu calculation of paving boundaries is time consuming and cannot meet the requirements of field engineering. There is an urgent requirement for an optimization algorithm to meet the recognition accuracy of paving boundaries in complex environments and significantly improve the calculation time.

4- Using complex mathematical equations does not guarantee the acceptance of a paper. Reduce them and use them alongside some illustrations to make the paper readable by not specialists.
Answer：Thank you very much for your comments. According to your constructive opinion, we delete the formula of the second half of the HHO algorithm, leaving only the parts that need to be optimized and modified. (manuscript page 3-8). 


According to your requirements for construction, we have added a section (flowchart of Otsu threshold optimized by enhanced HHO) for better reading and understanding. (manuscript page 10-11).
5) Flowchart of Otsu thresholding optimized by enhanced HHO
Fig.1 is the flowchart of Otsu thresholding optimized by enhanced HHO. Through the flowchart, we can quickly understand the calculation process and the specific location of optimization.


Fig.1 Flowchart of Otsu Thresholding Optimized by Enhanced HHO.


5- Quality of the illustrations are low. Not acceptable for a paper for journals
Answer：Thank you very much for your comments. The quality of the picture may be because there is no good typesetting when producing PDF. I uploaded pictures in other formats again. Please download the original picture again.

  


6- Figure 2. I can’t read and see what are the results and the differences. Only blue and red lines are visible.

Answer：Thank you very much for your comments. The red line is the standard deviation(STD) and the black line is the average of results(AVG). According to your constructive comments, we add the following description to the original description of Fig. 1 and Fig.2 respectively. (manuscript page 13-14).
The optimal solutions of F1–F7 are all 0, so the smaller the value, the better. The red line is the standard deviation and the black line is the average.
Combined with fmin in Table 4, PTSHHO can achieve the best results except F8, F12 and F13.
7- Figure 3. Convergence history should be visible.
7-图3。收敛的趋势应该是可见的
Answer：Thank you very much for your comments. According to the constructive opinions of you and other experts, we add the comparison with AHA and GNDO respectively in the convergence curve, so as to better express the convergence history. (manuscript page 14-16).
In this section, we discuss certain phenomena observed during the convergence behavior of PTSHHO for the UM and MM problems. The convergence behavior is in the form of a curve, and it directly reflects the convergence speed and accuracy.

 
Fig.4 Convergence behavior for unimodal problems.
Fig. 4 shows the convergence behavior for the UM problems and the average results of the 20 tests. It is worth noting that in order to make GNDO converge more easily, F1-F7 corresponding to GNDO in Fig.4 are the test data when dim = 2, and the convergence curves of other algorithms are tested when dim = 30. Fig.4 shows that PTSHHO is more stable than AHA and GNDO. There will be no problem of falling into local optimization in unimodal test function, which is more in line with the application of on-site complex environment. PTSHHO and TSHHO converge considerably faster for F1–F4. In addition, PTSHHO has higher accuracy, particularly for F3. The convergence speed of PTSHHO is relatively fast but not evident for F5–F7. The convergence accuracy of PTSHHO is slightly better for F5–F6 but not for F7. In summary, PTSHHO shows a higher convergence speed for the UM problems and better convergence accuracy in most cases.

In the case of the MM problems, there is a gratifying phenomenon in the convergence behavior of PTSHHO. The results for the three MM problems shown in Fig. 5 are unique. All algorithms except PTSHHO fall into local optimal solutions. As the convergence accuracy varies significantly compared to the previous part of the convergence curve, PTSHHO has a higher convergence speed.


Fig.5 Convergence behavior for multimodal problems (F21-F23).
The following conclusions can be drawn from the convergence curves for the UM and MM problems. PTSHHO provides a higher convergence speed. The number of iterations can be reduced for different problems. This can effectively reduce the calculation time to meet the time requirements of engineering projects.


8- Why did you bring pseudo code after the results? The paper should be organized properly
Answer：According to your construction suggestions, we adjusted the pseudo code to 3.2 the proposed HHO enhanced by tanh and PSO, so as to better explain the implementation of the algorithm. (manuscript page 9-10). 


.


4) Pseudocode of Otsu thresholding optimized by enhanced HHO
The pseudo code of Otsu Thresholding Optimized by Enhanced HHO is shown in table 2. The specific code is in data availability.
Table 2. Pseudocode.
Pseudo code of Otsu Thresholding Optimized by Enhanced HHO
Inputs: The population size N , maximum number of iterations T and image Outputs: The location of rabbit and its fitness value(variance) Use sine mapping to initialize the Population  （ Eq.(12)） while (stopping condition is not met) do      Calculate the fitness values of hawks      Set Xrabbit as the location of rabbit (best location)      Calculate the probability of gray value       for (each hawk (Xi)) do          Update the initial energy E0 and jump strength J            E0=2rand()-1,          J=2(1-rand())          Update the E using Eq. (11)          E=2*(1-4*tanh(t/T)/pi)*E0          if (|E|≥ 1)             Update the location vector using Eq. (5) And Eq. (9)             if (|E|< 1)                if (r ≥0.5 and |E|≥ 0.5 )                  Update the location vector                else if (r ≥0.5 and |E|< 0.5 )                  Update the location vector                 else if (r < 0.5 and |E|≥ 0.5 )                  Update the location vector                 else if (r < 0.5 and |E|< 0.5 )                  Update the location vector  Return Xrabbit Return the location of rabbit and its fitness value


9- Since the results of the proposed algorithm are not compared to the results of other algorithms, other well-known metaheuristics should be used for having a complete comparative investigation should be conducted by consideration of the results of other recently proposed metaheuristic algorithms.
Answer：Thank you very much for your comments. According to your suggestions, we compare and analyze the results of PTSHHO, AHA and GNDO. It shows that PTSHHO is more suitable for complex and changeable sites and can converge well in most cases. This part is revised in combination with recommendation 7. The final revision is as follows.
In this section, we discuss certain phenomena observed during the convergence behavior of PTSHHO for the UM and MM problems. The convergence behavior is in the form of a curve, and it directly reflects the convergence speed and accuracy.
 
Fig.4 Convergence behavior for unimodal problems.
Fig. 4 shows the convergence behavior for the UM problems and the average results of the 20 tests. It is worth noting that in order to make GNDO converge more easily, F1-F7 corresponding to GNDO in Fig.4 are the test data when dim = 2, and the convergence curves of other algorithms are tested when dim = 30. Fig.4 shows that PTSHHO is more stable than AHA and GNDO. There will be no problem of falling into local optimization in unimodal test function, which is more in line with the application of on-site complex environment. PTSHHO and TSHHO converge considerably faster for F1–F4. In addition, PTSHHO has higher accuracy, particularly for F3. The convergence speed of PTSHHO is relatively fast but not evident for F5–F7. The convergence accuracy of PTSHHO is slightly better for F5–F6 but not for F7. In summary, PTSHHO shows a higher convergence speed for the UM problems and better convergence accuracy in most cases.
In the case of the MM problems, there is a gratifying phenomenon in the convergence behavior of PTSHHO. The results for the three MM problems shown in Fig. 5 are unique. All algorithms except PTSHHO fall into local optimal solutions. As the convergence accuracy varies significantly compared to the previous part of the convergence curve, PTSHHO has a higher convergence speed.


Fig.5 Convergence behavior for multimodal problems (F21-F23).
The following conclusions can be drawn from the convergence curves for the UM and MM problems. PTSHHO provides a higher convergence speed. The number of iterations can be reduced for different problems. This can effectively reduce the calculation time to meet the time requirements of engineering projects.
10- The optimization problem statement in this paper is not in an acceptable level for a journal paper. A new section for this purpose is required.
10-本文中的优化问题陈述不符合期刊论文的可接受水平。为此需要一个新的章节。
Answer：Thank you very much for your comments. According to your construction suggestions, we have added a new chapter engineering optimization problems to explain the on-site problems that need to be optimized in this paper(manuscript page3). 

2. Engineering optimization problems
This study focuses on the detection of paving boundaries in the core wall area of earth-rock dams. Images are mainly obtained using the monitoring cameras on the dam abutments on both banks. The conventional Otsu calculation of paving boundaries is time consuming and cannot meet the requirements of field engineering. There is an urgent requirement for an optimization algorithm to meet the recognition accuracy of paving boundaries in complex environments and significantly improve the calculation time.
11- The conclusion and the Abstract of the paper lacks the main quantitative results of this manuscript. It should be noted that some numerical results of the paper should be mentioned properly in the conclusion section.

Answer：Thank you very much for your comments. According to your constructive questions, quantitative results are added to the conclusion of this paper(manuscript page 19).
This study optimizes the HHO algorithm, which significantly improves its accuracy and convergence speed. The universality, superiority, and consistency of PTSHHO are verified through multiple UM and MM test problems. In addition, PTSHHO and Otsu are combined to rapidly detect the boundaries of materials in high-definition images. Finally, the proposed method is applied to engineering data, and it achieves excellent results. The method is applied to a real-life project, where it reduces the calculation time to 20 s, which is approximately 18.8% of the original calculation time.

The proposed method can also be to other projects of the same type. Similar engineering application scenarios, such as roads and airports, must be verified in future engineering practice. In future work, other application scenarios for optimized PTSHHO can be developed and combined with other algorithms.

12- Number of the Objective Function Evaluation (OFE) in the utilized methods and the modified version alongside the other methods in the revision should be provided in separate table for comparative purposes.
Answer：Thank you very much for your comments. At the end of this paper, an appendix is added to explain the specific expression of the test function. 
Appendix. Benchmark Functions of Table 1
1. 
2. 
3. 
4. 
5. 
6. 
7. 
Appendix. Benchmark Functions of Table 2
1. 
2. 
3. 
4. 
5. 
6. 
7. 
8. 
9. 
10. 
11. 
12. 
13. 
14. 
15. 
16. 
13- Improve the English level of the paper and correct the typos.

Answer：Thank you very much for your comments. We are so sorry for the poor English expression. In order to make the article easier to understand, the language of the revised manuscript has been improved by the American Journal Experts and Elsevier. The editorial certificate of our paper and the revision are shown below. See revised manuscript with track changes for details.


Reviewer #2：
1- I suggest you combine the introduction and literature review parts. Also, the literature review part needs to be enriched with more references in … In recent years, many swarm optimization algorithms have been proposed and widely applied, such as particle swarm optimization [30], grey wolf optimizer [31]and Harris hawks optimizer [14]. Heidari et al. [14] compared HHO with other algorithms and demonstrated its advantages. HHO has proven its superiority in many applications [32-36], I suggest the following [1-3]:

• [1]. Reliability analysis based improved directional simulation using Harris Hawks optimization algorithm for engineering systems. Engineering Failure Analysis, 135, 106148. doi.org/10.1016/j.engfailanal.2022.106148
• [2]. First-order reliability method based on Harris Hawks Optimization for high-dimensional reliability analysis. Structural and Multidisciplinary Optimization, 62(4), 1951-1968. doi.org/10.1007/s00158-020-02587-3
• [3]. Using particle swarm optimization algorithm to optimally locating and controlling of pressure reducing valves for leakage minimization in water distribution systems. Sustainable Water Resources Management, 6(4), 1-11. doi.org/10.1007/s40899-020-00426-3
Answer：Thank you very much for your comments. It has been revised according to your suggestion.
[12] J. Jafari-Asl, B. Sami Kashkooli, M. Bahrami, Using particle swarm optimization algorithm to optimally locating and controlling of pressure reducing valves for leakage minimization in water distribution systems, Sustainable Water Resources Management, 6 (2020) 64.
[15] J. Jafari-Asl, M.E.A. Ben Seghier, S. Ohadi, J. Correia, J. Barroso, Reliability analysis based improved directional simulation using Harris Hawks optimization algorithm for engineering systems, Engineering Failure Analysis, 135 (2022) 106148.
[16] C. Zhong, M. Wang, C. Dang, W. Ke, S. Guo, First-order reliability method based on Harris Hawks Optimization for high-dimensional reliability analysis, Structural and Multidisciplinary Optimization, 62 (2020) 1951-1968.

2- I would suggest showing the methodological approach through a flowchart.
Answer：Thank you very much for your comments. It has been revised according to your suggestion. According to your requirements for construction, we have added a section (flowchart of Otsu threshold optimized by enhanced HHO) for better reading and understanding. (manuscript page 10-11).
5) Flowchart of Otsu thresholding optimized by enhanced HHO
Fig.1 is the flowchart of Otsu thresholding optimized by enhanced HHO. Through the flowchart, we can quickly understand the calculation process and the specific location of optimization.

Fig.1 Flowchart of Otsu Thresholding Optimized by Enhanced HHO.

3- Figs. 3 and 4 show the convergence curves of variant version of HHO. You can view the results of new algorithms such as AHA [4] or GNDO [5] (and etc.) and compare them with the results of your algorithm.
[1]. Artificial hummingbird algorithm: A new bio-inspired optimizer with its engineering applications. Computer Methods in Applied Mechanics and Engineering, 388, 114194. doi.org/10.1016/j.cma.2021.114194
[2]. Generalized normal distribution optimization and its applications in parameter extraction of photovoltaic models. Energy Conversion and Management, 224, 113301. doi.org/10.1016/j.enconman.2020.113301
Answer：Thank you very much for your comments. It has been revised according to your suggestion. According to your constructive suggestions, PTSHHO algorithm is comprehensively compared with AHA and GNDO algorithms.
3.3.2 Convergence curve of PTSHHO
In this section, we discuss certain phenomena observed during the convergence behavior of PTSHHO for the UM and MM problems. The convergence behavior is in the form of a curve, and it directly reflects the convergence speed and accuracy.

 
Fig.4 Convergence behavior for unimodal problems.
Fig. 4 shows the convergence behavior for the UM problems and the average results of the 20 tests. It is worth noting that in order to make GNDO converge more easily, F1-F7 corresponding to GNDO in Fig.4 are the test data when dim = 2, and the convergence curves of other algorithms are tested when dim = 30. Fig.4 shows that PTSHHO is more stable than AHA and GNDO. There will be no problem of falling into local optimization in unimodal test function, which is more in line with the application of on-site complex environment. PTSHHO and TSHHO converge considerably faster for F1–F4. In addition, PTSHHO has higher accuracy, particularly for F3. The convergence speed of PTSHHO is relatively fast but not evident for F5–F7. The convergence accuracy of PTSHHO is slightly better for F5–F6 but not for F7. In summary, PTSHHO shows a higher convergence speed for the UM problems and better convergence accuracy in most cases.

In the case of the MM problems, there is a gratifying phenomenon in the convergence behavior of PTSHHO. The results for the three MM problems shown in Fig. 5 are unique. All algorithms except PTSHHO fall into local optimal solutions. As the convergence accuracy varies significantly compared to the previous part of the convergence curve, PTSHHO has a higher convergence speed.

 
Fig.5 Convergence behavior for multimodal problems (F21-F23).
The following conclusions can be drawn from the convergence curves for the UM and MM problems. PTSHHO provides a higher convergence speed. The number of iterations can be reduced for different problems. This can effectively reduce the calculation time to meet the time requirements of engineering projects.


4- In section 4, more details about case study is needed.
Answer：Thank you very much for your comments. It has been revised according to your suggestion. According to your constructive suggestions, we have supplemented and modified parts 4.1 and 4.3. (manuscript page 17-19). 
Fig.7 shows the entire process of the basic method, from the paving process to quality acceptance. First, the zone surface information to be rolled is extracted from the earth rock dam rolling real-time monitoring system developed by our research group [52]. Second, the corresponding image is obtained from the dataset collected by the camera.Finally, the paving boundary in the target area is identified and compared with the design paving boundary read by the paving system. If paving encroachment occurs, it is fed back to the corresponding onsite personnel according to different alarm levels, and they provide feedback.

4.3 Field application
Engineering data are collected from LHK, and the system based on PTSHHO-Otsu is applied to the project. This effectively reduces the labor cost and time required for the corresponding processes.
Fig. 9 shows the system applied at the project site and the paving results of the zone surface. The coarse aggregate occupies the core wall zone, and a first-class early warning is generated when the zone area is more than 4 m2. A level II early warning is generated when the zone area is less than 4 m2 but more than 1 m2. A level III early warning is generated when the zone area is less than 1 m2.
In addition, the operating speed of the system is approximately 20 s, which fully meets the construction requirements of the project site. The online operation of the system has been highly praised by construction sites because of its significant practical engineering value and suitability for other engineering fields.


5- Conclusion has to be improved. 
Answer：Thank you very much for your comments. It has been revised according to your suggestion According to your constructive suggestions, we have supplemented and modified parts5. (manuscript page 19-20). 
This study optimizes the HHO algorithm, which significantly improves its accuracy and convergence speed. The universality, superiority, and consistency of PTSHHO are verified through multiple UM and MM test problems. In addition, PTSHHO and Otsu are combined to rapidly detect the boundaries of materials in high-definition images. Finally, the proposed method is applied to engineering data, and it achieves excellent results. The method is applied to a real-life project, where it reduces the calculation time to 20 s, which is approximately 18.8% of the original calculation time.

The proposed method can also be to other projects of the same type. Similar engineering application scenarios, such as roads and airports, must be verified in future engineering practice. In future work, other application scenarios for optimized PTSHHO can be developed and combined with other algorithms.
